# Transforming transfusion safety: Insights from implementing bedside electronic checks at a large UK National Health Service trust

**DOI:** 10.1111/vox.70189

**Published:** 2026-02-22

**Authors:** Florence Oyekan, Montasir Ahmed, Catherine Booth, Louise Bowles, Ollie Djurdjevic, Yan Feng, Claudio Geraci, Laura Green, Kirsty Hancock, Suzanne Makki, Helinor McAleese, Michael F. Murphy, Nathan Proudlove, Josephine McCullagh

**Affiliations:** ^1^ Barts Health NHS Trust London UK; ^2^ Blizard Institute, Barts and the London School of Medicine and Dentistry Queen Mary University of London London UK; ^3^ Wolfson Institute of Population Health Queen Mary University of London London UK; ^4^ NHS Blood and Transplant London UK; ^5^ Radcliffe Department of Medicine University of Oxford Oxford UK; ^6^ Oxford University Hospitals NHS Trust Oxford UK; ^7^ Alliance Manchester Business School University of Manchester Manchester UK

**Keywords:** bedside electronic transfusion checks (BETC), clinical workflow integration, healthcare technology implementation, quality improvement, staff training and engagement, transfusion safety

## Abstract

**Background and Objectives:**

Bedside electronic transfusion checks (BETC) enhance transfusion safety by reducing errors associated with manual processes. Despite national recommendations, BETC adoption in the United Kingdom remains limited. This study reports on the implementation of BETC at four hospitals at Barts Health NHS Trust, aiming to share insights on the implementation process.

**Materials and Methods:**

The main implementation was split into three phases: (1) pre‐pilot, (2) pilot and (3) main implementation (2022–2025). Staff surveys on training satisfaction and key performance indicators (KPIs) on transfusion activity were used to evaluate the uptake of the BETC system. Statistical process control (SPC) charts were used to identify trends, variation and patterns in the data following the implementation of BETC.

**Results:**

A total of 5079 staff were trained and 404 personal digital assistant (PDA) devices deployed across four hospitals. Early implementation highlighted that training 60% of staff was insufficient for optimal system use, increasing this threshold to 80% improved adoption. BETC was initially more commonly used for blood administration than group and screen (G&S) sample labelling. Over time, increased usage of BETC for G&S labelling correlated with a marked reduction in sample rejection rates across all sites. Staff reported high satisfaction with training, with 99.5% rating it positively.

**Conclusion:**

Early adopters played a pivotal role, but achieving widespread adoption required extended training and support. Addressing technical and workflow barriers, coupled with mandatory system use, could enhance the speed of impact of BETC. These insights offer guidance for future adopters aiming to improve transfusion safety and efficiency.

**Highlights**

- For the implementation of transfusion safety measures, stakeholder engagement and information technology integration were found to be key.
- Staff preferred using bedside electronic transfusion checks for blood administration over group and screen labelling initially.
- A phased rollout enabled iterative improvements and sustained adoption.

## INTRODUCTION

The implementation of bedside electronic transfusion checks (BETC) is an important advancement in improving the safety and efficiency of blood transfusion practice. Reports from the UK haemovigilance scheme, Serious Hazards of Transfusion (SHOT), show that errors, such as the administration of the incorrect blood component or mislabelling group and screen (G&S) samples, remain leading causes of transfusion‐related adverse events [1]. These errors are often attributable to the complexity of manual processes and lapses in attention during the positive patient identification (ID) process [2, 3].

The UK Infected Blood Inquiry highlighted systemic failures in transfusion safety, resulting in devastating consequences to patients and their families. It called for stronger safety protocols and technological advancements to prevent future harm [4, 5]. By incorporating real‐time bedside checks using barcode scanning, BETC has the potential to ensure that the right blood component is given to the right patient, enhancing safety and improving patient outcomes.

The introduction of electronic identification systems has been one of the main recommendations issued by SHOT [6], the UK National Patient Safety Agency (NPSA) [7] and UK national guidelines [8] to reduce human error and improve transfusion safety. Despite these recommendations, the adoption of BETC in the United Kingdom has been poor [9]. Successful deployment requires significant capital investment, robust information technology (IT) infrastructure and ongoing training and maintenance of the system [10, 11]. Furthermore, the alignment of technical systems across hospital departments is critical for ensuring the seamless operation of these systems [12, 13].

This paper reports the experience of introducing BETC at a large UK NHS trust (Barts Health NHS Trust) exploring the successes and challenges encountered. We report on three key areas of the implementation project: stakeholder engagement, technical (digital and organizational) and staff training. By sharing these insights, we aim to guide future adopters in navigating the complexities of implementation while maximizing the full potential of this technology to improve transfusion safety and patient outcomes.

## MATERIALS AND METHODS

### Organizational structure

Barts Health Trust (BH) is comprised of four hospitals; for this paper, they will be referred to as Hospital 1 (H1), Hospital 2 (H2), Hospital 3 (H3) and Hospital 4 (H4). The Trust has one of the largest blood transfusion services in the country, administering over 65,000 units to approximately 15,000 patients per year. The Trust is home to the largest trauma centre in the country, provides specialist cardiac surgery and oncology services and manages a large cohort of haemoglobinopathy patients who require blood transfusion support. Three of the hospitals have emergency department services and provide general medical and surgical services to adults and paediatric patients.

The Trust has an electronic patient record (EPR) system (Cerner Millennium) and electronic prescribing for blood components. Transfusion laboratories are located on each hospital site and equipped with a laboratory information management system (LIMS) (Clinysis, Winpath Enterprise) integrated across the sites, fully automated testing and electronic fridges (Haemontics, BloodTrack Tx) in the laboratory and clinical areas, which enable the remote electronic issue of components.

### Implementation of BETC

#### Funding

The implementation of BETC was funded by Barts Charity and NHS Charities Together as a service improvement project for the Trust.

#### BETC system

The BETC system implemented was BloodTrack Tx provided by Haemontics. The system consists of Zebra TC‐52 personal digital assistants (PDAs) with an Android operating system, Zebra mobile printers and BloodTrack Tx software. The PDAs were designed to enable users to scan their own ID (confirming who performed the task), a patient's wristband (checking patient's details) and new 2 dimensional (2D) barcodes on blood component compatibility labels (checking unit's details). Additionally, the mobile printers generate G&S sample labels to be attached directly to the blood sample tube.

#### Implementation

To ensure the successful implementation of BETC, a dedicated specialist project team was assembled. The team included a project manager, an IT specialist and two specialist transfusion practitioners (TPs), one with a nursing background and one with a transfusion science background [14, 15]. The team provided training and guidance on transfusion‐related workflows, and managed system integration and technical challenges. A separate governance structure was also developed to oversee the delivery of the project.

Transfusion training of all clinical staff is an integral part of the TP role [14, 15]. To ensure consistency and to support integration into routine practice, training was mainly delivered by the project's dedicated TPs and supported by hospital‐based TPs. Training was delivered either face to face or online, and competency assessments were completed for both the blood administration and sample collection processes. Initially, a target of 60% of all staff trained per hospital was agreed before the project team moved to the implementation of BETC at the next hospital; following this, the use of BETC was closely monitored by the project team and refresher training was offered to staff who required an update.

The project adopted a phased implementation strategy over a 3‐year period from April 2022 to March 2025 covering all inpatient clinical areas and haematology day units (HDUs). The G&S sampling in outpatient departments was excluded at this stage and is scheduled as a separate project. The implementation was split into three phases: (1) pre‐pilot, (2) pilot and (3) main implementation [16].

1. *Pre‐pilot*: Prior to piloting the devices, time was spent preparing the sites for the implementation of BETC. This preparation involved developing a detailed ‘as‐was’ process map of the existing end‐to‐end transfusion pathway, highlighting areas that would require changes to accommodate the new system. Laboratory and clinical documents were updated to reflect any changes in practice.
2. *Pilot*: The pilot was performed in the HDUs at H1 and H3. The pilot aimed to provide a controlled environment to evaluate the implementation of BETC and allow the project team to identify and resolve any unforeseen issues that had not been picked up in the pre‐pilot work. The HDUs at both H1 and H3 were chosen because of pre‐planned and high‐volume transfusion performed in these areas.
3. *Main implementation*: The main system roll‐out followed a phased approach. When 60% of the staff were trained and had submitted the competency assessment, devices were released to the wards for use. After completion of the roll‐out in one hospital site, a 3‐week evaluation period was introduced before implementation of the new system was moved to the next hospital.

Key performance indicators (KPIs) were established and used to monitor the progress of implementation throughout the project. These were the proportion of blood components transfused using BETC and the proportion of G&S rejections. G&S sample rejections were defined as any sample that was rejected due to mislabelling or errors in any of the core patient identifiers (first name, surname, date of birth, hospital/NHS number). Other reasons for sample rejection were not included in the analysis.

#### Staff survey

An anonymous electronic survey targeted at clinicians involved in G&S sample labelling or blood administration (nurses, doctors and support teams such as healthcare assistants) was developed using Microsoft Forms to assess staff satisfaction with the training provided and to allow staff to comment on areas where improvements might be required. Respondents were asked to rank their satisfaction with training on a scale of 1–10, with 1 being very dissatisfied, 5 being neutral and 10 being very satisfied. The project received Research Ethics Approval (No. 22/NW/0138) and Health Research Authority approval for data collection (IRAS No. 311676).

### Data analysis

The data were analysed using descriptive statistics and narrative analysis to provide a comprehensive overview of the challenges and facilitators to implementing BETC. Statistical process control (SPC) charts, commonly used in quality improvement projects [17, 18, 19], were used to highlight changes in the system post implementation.

## RESULTS

Key challenges and facilitators encountered during each phase of implementation (pre‐pilot, pilot and main implementation) can be grouped broadly into three categories: (1) technical, (2) training and (3) stakeholder engagement (Table 1), and are reported separately for each phase of implementation.

**TABLE 1:** Overview of common challenges and facilitators to implementation of bedside electronic transfusion checks.

Themes	Challenges or facilitators	Solutions/recommendations
Technical—Digital	Duplication of workflows (vital signs on PDAs not integrated with EPR); Issues with messaging between LIMS and BETC; Device connectivity and updates; Neonatal wristbands did not have a barcode, as the wristband was too small to fit both the barcode required for the PDA and other barcodes. Wristband printers in clinical areas were not compatible with barcode formats required by the new devices.	Removal of duplicated steps on PDAs; Regular meetings with supplier to resolve any functional issues; Dedicated IT support and future plans to add devices to MDM; On‐going discussions with Trust IT team to resolve: Medium‐term solution: to have two wristbands for neonates. Long‐term solution: redesign the neonatal wristband Trust IT upgraded the ward printer software to be compatible with new barcodes.
Technical—Organizational	Clinical ward relocation in one hospital during implementation for operational purposes; Emergency blood transfusion function on the PDAs did not function correctly with the existing emergency transfusion workflows.	Careful planning and discussion will all teams involved to reduce the impact on the implementation of the PDAs; Close engagement with emergency department and theatres—Emergency workflows adapted.
Training	Competency assessment document overly complicated; Delays in obtaining competency of trained staff; Efficient and well‐received training delivered by the TPs.	Changes made to simplify the document; All trained staff were asked to complete the competency immediately after training sessions; The number of trainees per session was reduced to no more than 5 per trainer. N/A
Stakeholder engagement	Governance structures to disseminate information and support on‐going challenges TP team creativity and resourcefulness.	N/A N/A

Abbreviations: BETC, bedside electronic transfusion checks; EPR, electronic patient record; LIMS, laboratory information management system; IT, information technology; MDM, mobile device management; N/A, not applicable; PDA, personal digital assistant; TP, transfusion practitioner.

### Pre‐pilot

The pre‐pilot stage focused on completion of all prerequisites to allow for the end‐to‐end usability of the new system. Items that had to be completed are described in Table 2, prior to starting the training of clinical staff. Key challenges during this phase were mainly technical, both digital and organizational. Digital challenges were mainly due to the wristband printers in clinical areas not being compatible with the barcode formats required by the PDAs. A solution was found after working with the Trust IT team to upgrade the software of the current ward printers. This resulted in significant cost savings for the Trust, as there was no need to purchase new printers for the wards. Organizational challenges mainly focused on staffing issues and resource management due to major regional pathology restructuring.

**TABLE 2:** Prerequisites to be completed.

1. BloodTrack Tx—Pre‐installation
Confirm PDA and printer requirements
Confirm wristband barcode type
Provide wristband barcode and confirm all sites will be the same
Test 2D barcode compatibility
Confirm wristband 2D barcode is compatible
Confirm if 2D barcode is available on compatibility label
Confirm 2D label requirements
Create label
Confirm wireless requirements (i.e., coverage, etc.)
2. System configuration and project team training
Confirm device management type (MDM, etc.)
Add devices to network
Deploy software to devices
Lock devices down to transfusion only
Configure initial PDAs with server
Test initial PDA/server connectivity
Initial PDA installation/configuration
PDA installation/configuration training
Initial printer installation/configuration
Printer installation/configuration Training
Configuration/profile review (IT)
Configuration/profile review (clinical)
Finalized profile configuration ready for validation
Configuration document produced
3. Initial BloodTrack Tx and compatibility validation
PDA validation script to be created
PDA validation script signed off
Compatibility label validation script to be created
Compatibility label validation script signed off
PDA validation
Compatibility label validation
PDA configuration and change board sign‐off
4. Pilot BloodTrack Tx pilot training
End‐user training (pilot specific)
Add trained users to BloodTrack
5. BloodTrack Tx Go‐Live (prepare for pilot stage—Haematology day units at H1 and H3)
Go live on BloodTrack at both hospitals
Pilot testing and validation complete
Identify power points/locations to dock PDAs
Instal PDAs on ward and test connectivity
Configure compatibility label and printer
Completed supervisor/trainer training documents
Add trained users to ASK
6. BloodTrack Tx rollout—(Prepare for full roll‐out) go live
Purchase PDAs and label printers
Instal PDAs/printers in all locations
Confirm wireless connectivity and configuration in all locations
Finalize all training documents and videos for end‐user training—All wards

Abbreviations: 2D, 2 dimensional; H, hospital; IT, information technology; MDM, mobile device management; PDA, personal digital assistant.

### Pilot

A total of 47 staff members were trained on the use of BETC on the pilot wards in April and May 2023. The uptake of the devices in the pilot wards was very quick, with all the devices being used appropriately by all staff. The main challenges seen at this stage were broadly related to IT issues, which included problems with electronic messages not being transferred from BETC to LIMS and issues with printing 2D barcodes on either patient wristbands or blood compatibility labels. When asked to provide feedback on how the system could be improved, the main comments were related to streamlining the process and reducing duplication, for example, removing the requirement to submit vital signs on the PDAs, as these were already submitted by nurses via the EPR (Table 1).

### Main implementation

The main implementation (i.e., training of clinical staff) commenced with H1 (June–November 2023 inclusive), followed by H2 (November 2023–March 2024 inclusive), H3 (March–October 2024 inclusive) and finally H4 (October–April 2025 inclusive). This included a 3‐week evaluation period for each site.

A total of 404 PDA kits were rolled out across the Trust (H1 78, H2 56, H3 180 and H4 90) and 5079 staff were trained (H1 940, H2 970, H3 2074 and H4 1095). Very early in the implementation period for H1, we noticed that staff were not submitting the training competency on time, resulting in the project team not releasing the devices to the wards, as less than the required 60% of their staff were trained and had completed their competencies. As a result, for subsequent hospitals the decision was made to perform staff competency assessment immediately after the training and that a training session must have a maximum of only five people to enable competency assessment at the time and to allow for more constructive feedback. This approach improved the timeliness of device release to the clinical wards for these other hospitals.

**FIGURE 1:**
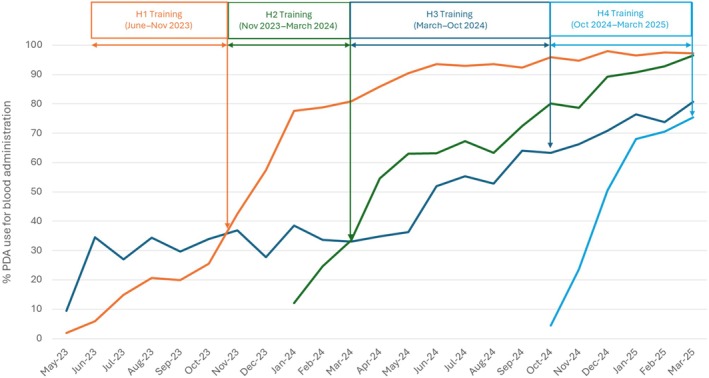
Proportion of blood components administered using bedside electronic transfusion checks (BETC) personal digital assistants (PDAs) at four hospitals from May 2023 to March 2025. Training periods are indicated for all four hospitals. H, hospital.

However, we noticed that, despite 60% of staff being trained to use the devices, BETC was still not being used to its full potential by the end of each training period (Figure 1); so, a decision was made for some of the project team to continue supporting each site with training to achieve a target level of 80% of all staff trained in each clinical area. This increased the uptake of the devices over time, and at 12 months after the implementation, H1 and H2 had both achieved 90% usage of BETC devices for blood administration.

**FIGURE 2:**
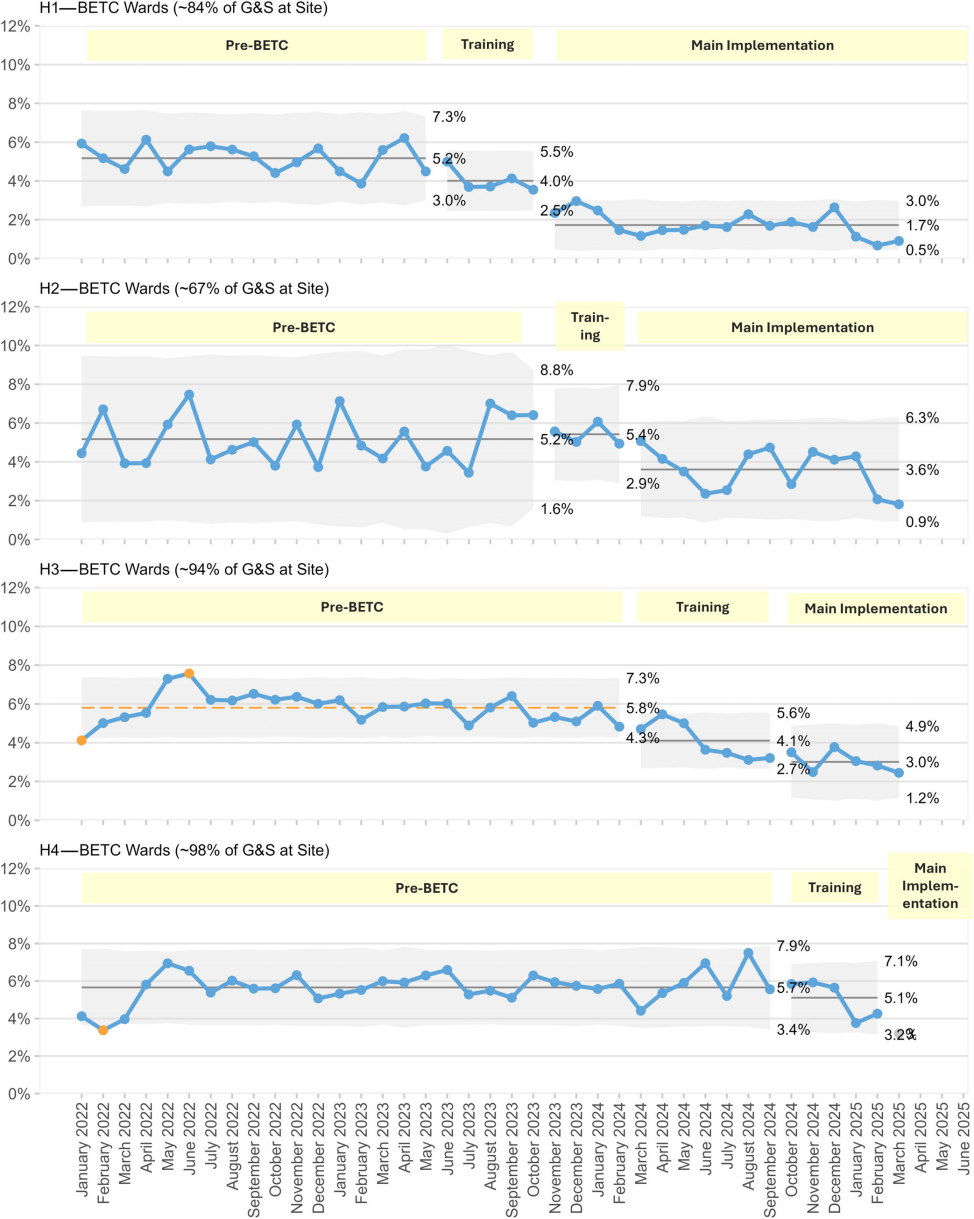
Statistical process control (SPC P′) charts of group and screen (G&S) sampling episode rejection rates for pre‐bedside electronic transfusion checks (BETC) mislabelling reasons, split into epochs: Pre‐BETC, BETC training, main implementation. The middle of each set of three percentages is the mean. The other percentages, and the grey bands, are the usual SPC mean ± 3 sigma process limits (the expected range of random variation). Orange datapoints are outside this range, so potentially unusual values; there are no issues of concern with these here.

The other pattern we noticed was that staff were initially more likely to use the devices for blood administration than for G&S sample labelling. Therefore, the impact of BETC usage on G&S sample rejection rate was slow to develop. As the uptake of BETC devices for G&S sample labelling increased, the rate of G&S sample rejection decreased, an effect we saw across all four hospitals (Figure 2).

### Feedback on training

A total of 2084 respondents completed the survey on staff satisfaction with the training provided (a response rate of 33%). Most of the respondents were nurses 1687 (81%), followed by doctors 239 (12%) and healthcare support staff 158 (8%). Only two staff members reported having received training online; most responders received face‐to‐face training. Reported training session duration varied from 15 min (*n* = 264, 12.5%) to >1 h (*n* = 98, 4.5%), depending on the size of the groups, with most of the training sessions being 30–60 min (*n* = 1743, 82.8%). Overall, training feedback was positive, with 99.5% rating the training at 5 or above. Additionally, 89.3% (*n* = 1861) of staff members indicated that they required no further training, with the others expressing a desire for additional training in order to feel fully comfortable using the device.

## DISCUSSION

The implementation of BETC at four hospitals in the Trust provides key insights into the adoption of safety‐focused transfusion technology. The integration of BETC addressed systemic inefficiencies and human error risks in transfusion processes, aligning with recommendations from SHOT and other national bodies. While implementation yielded significant improvements in KPIs, the process highlighted the complexity of integrating new systems into large, diverse healthcare organizations.

The adoption of BETC in our case can be examined using the Early Adopters Theory from Rogers' seminal diffusion of innovation model [20, 21]. Early adopters (key clinical staff and departmental leaders) played a pivotal role in championing the system during pilot phases. Their engagement helped generate initial buy‐in and facilitated system uptake among broader user groups. However, achieving the 60% training target, as previously shown by Murphy et al [2], proved insufficient. Our results also show that later adopters required more robust engagement and extended support, and this aligns with the theory, which emphasizes the need for tailored strategies to address varying levels of readiness and scepticism across adopter categories.

Increasing the training threshold to achieve 80% staff coverage before declaring ‘Business‐As‐Usual’ status was a critical decision that accelerated device adoption and system utilization. The phased implementation also allowed the project team to address laggards (individuals or groups resistant to change) by maintaining on‐site presence and offering additional resources to alleviate concerns. However, as depicted in both Figures 1 and 2, there remains a gap between the current uptake of the system and the system being used to its full potential (100% usage for administration of transfusion, and 0% G&S rejections for mislabelled samples).

One of the project's key successes was having a proactive stakeholder engagement strategy at a very senior level, which ensured downstream engagement of all staff and regular feedback loops. These mechanisms facilitated interdepartmental collaboration, enabling the resolution of challenges such as device compatibility and workflow integration.

Training was another key success of the implementation strategy. Having face‐to‐face training by experienced TPs ensured clinical relevance, optimized training delivery, allowed the issue of competency certification immediately after the training sessions and also enabled users to ask further questions immediately after the training. Providing training for rotational staff, such as junior doctors and staff on different shift patterns, and freeing up staff in busy clinical areas were challenging. Feedback reflected this by highlighting the need for simplified competency‐assessment documents and more adaptable training methods such as videos. We were unable to implement different methods during this stage of implementation but future implementations should consider hybrid training models, incorporating online modules and simulation‐based learning to address these gaps.

Despite its successes, the implementation faced several technical and organizational challenges. Device connectivity, barcode compatibility for smaller wristbands (like neonates) and the lack of integration with the EPR system created inefficiencies that slowed down the roll‐out. These issues highlight the importance of engaging IT and estates teams early in the planning process to address infrastructure and interoperability requirements. Moreover, the absence of mobile device management (MDM) for PDAs added to the workload of IT specialists, delaying updates and maintenance.

Post implementation, there needs to be continuous support and monitoring to ensure sustainability of system adoption [22]. The decision to extend training efforts and provide ongoing technical support ensured the gradual increase in device usage, achieving 95% adoption at some sites within 10 months. Systems such as BETC provide a more efficient and safer approach to transfusion practice; however, while use remains optional for staff rather than a mandatory part of the transfusion pathway, the systems will fail to reach 100% usage. Future plans in our hospitals will look to mandate the use of bedside electronic devices for both blood administration checks and G&S sample labelling, following an extensive risk assessment, weighing up the benefits of using the system to its full potential versus any potential risk to delaying transfusion care.

This study has several limitations that may impact the generalizability of its findings. First, the phased implementation was tailored to the specific organizational structure and resources of the Trust, which may not reflect the capabilities of smaller or less‐well‐resourced healthcare organizations; however, the mix of hospitals in our Trust allows for the lessons learnt from our implementation to be applied to a range of hospitals. Second, the reliance on face‐to‐face training sessions, while effective, posed logistical challenges for rotational staff (particularly night staff or junior doctors) and may not be scalable in larger implementations. Finally, the lack of integration with EPR and limited simulation‐based training hindered the system's initial functionality and user preparedness. Future implementations should prioritize seamless integration with existing IT systems, such as EPR, to reduce workflow duplication. Addressing infrastructure needs such as barcode functionality for all patient groups during the planning phase can also mitigate delays and enhance system usability.

In conclusion, key lessons highlighted from the implementation of the bedside electronic transfusion system at four hospitals in our Trust include the importance of early and continual stakeholder engagement, comprehensive training and adaptive project management to address unforeseen challenges. By adopting a phased implementation strategy and leveraging insights from early adopters, the project team successfully navigated the complexities of introducing innovative technology into a large healthcare trust.

Future adopters should prioritize pre‐implementation planning, including infrastructure assessments and IT system integrations, to streamline workflows and maximize impact. The inclusion of hybrid training models and simulation‐based learning can further enhance user readiness and confidence. Our experience highlights the critical role of adaptability, collaboration and iterative improvement in driving successful healthcare innovation.

## CONFLICT OF INTEREST STATEMENT

The authors declare no conflicts of interest.

## Data Availability

The data that support the findings of this study are available on request from the corresponding author. The data are not publicly available due to privacy or ethical restrictions.
